# Dimensional stability of 3D-printed models for orthodontic aligners: a six-month storage evaluation

**DOI:** 10.1590/2177-6709.31.1.e2625115.oar

**Published:** 2026-04-17

**Authors:** Paulo Mateus Pereira SOUSA, Karina Oliveira LUSTOSA, Dalila Mikaelly Ribeiro LUZ, Iara da Costa Araújo BARROS, Edson Ferreira DA SILVA, Alexandre Henrique de Melo SIMPLÍCIO, Wagner Leal DE MOURA, Marcus Vinicius Neiva Nunes DO REGO

**Affiliations:** 1 State University of Campinas, Dentistry Course, Department of Biosciences (Piracicaba/SP, Brazil).; 2 Federal University of Piauí, Dentistry Course (Teresina/PI, Brazil).; 3 State University of Campinas, Dentistry Course, Department of Public Health, Pediatric Dentistry, and Orthodontics (Piracicaba/SP, Brazil).; 4 Uninovafapi University Center, Dentistry Course, Department of Epidemiology and Etiology of Oral Diseases (Teresina/PI, Brazil).; 5 Federal University of Piauí, Dentistry Course, Department of Pathology and Dental Clinic (Teresina/PI, Brazil).

**Keywords:** Orthodontics, Clear aligners, 3D Printing, Ortodontia, Alinhadores transparentes, Impressão em 3D

## Abstract

**Objective:**

This study aimed to analyze the dimensional stability of 3D resin-printed models for orthodontic aligners under specific storage conditions over a period of up to six months.

**Methods:**

A model of the upper arch of a dental mannequin (Orbital Bone) was scanned using the 3Shape Trios 3 scanner, to create digital models (n = 20), which were printed on the Anycubic Photon X6 KS printer and stored in a dark box at room temperature for six months. Dimensional analysis was performed by comparing the meshes of the models through superimposition at the following time points: T0 - immediately after printing (control group), T1 - 1 month, T2 - 3 months, and T3 - 6 months of storage. The Ortho Analyser software was used for this purpose, and measurements were performed by a single, trained examiner. After confirming normality (p > 0.05, Shapiro-Wilk test), Analysis of Variance (ANOVA) was applied to verify dimensional changes in the following regions of the dental arch: incisors, canines, premolars, and molars.

**Results:**

Dimensional changes in the models were not statistically significant (p > 0.05) during the storage period for all evaluated regions. However, the greatest dimensional changes occurred during the first month and in the molar region (0.175 mm), which showed a tendency to expand.

**Conclusions:**

After six months of storage in a light-free environment, the 3D-printed models exhibited minor dimensional changes, which may have some clinical impact on aligner treatment, particularly in the molar region.

## RESUMO

**Objetivo:**

Este estudo objetivou analisar a estabilidade dimensional de modelos 3D impressos com resina para alinhadores ortodônticos, sob condições específicas de armazenamento, por um período de até seis meses.

**Material e Métodos:**

Um modelo da arcada superior de um manequim odontológico (Orbital Bone) foi digitalizado com um *scanner* 3Shape Trios 3 para criar modelos digitais (n=20), que foram produzidos na impressora Anycubic Photon X6 KS e armazenados em caixa escura por seis meses à temperatura ambiente. A análise dimensional foi realizada comparando-se, por meio de sobreposição, as malhas dos modelos nos tempos T0 - imediatamente após a impressão (grupo controle), T1 - 1 mês, T2 - 3 meses e T3 - 6 meses de armazenamento. Para tal, utilizou-se o *software* Ortho Analyser e as medições foram realizadas por um único examinador previamente treinado. Após confirmação de normalidade (p > 0,05, teste Shapiro-Wilk), utilizou-se a Análise de Variância (ANOVA) para verificar as alterações dimensionais nas seguintes regiões da arcada dentária: incisivos, caninos, pré-molares e molares.

**Resultados:**

As alterações dimensionais nos modelos não apresentaram significância estatística (p>0,05) durante o período de armazenamento para todas as regiões avaliadas. No entanto, as maiores mudanças dimensionais ocorreram no primeiro mês e na região dos molares (0,175mm), que apresentou uma tendência de expansão.

**Conclusão:**

Após seis meses de armazenamento em ambiente sem luz, os modelos 3D impressos apresentaram alterações dimensionais pequenas, mas que podem apresentar algum impacto clínico no tratamento com alinhadores, principalmente na região de molares.

## INTRODUCTION

The use of digital imaging and 3D printing has significantly impacted modern Dentistry and has become increasingly accessible and common in clinical practice. By converting digital information into physical models, this technology has expanded the range of available dental treatment options, offering greater predictability and comfort for patients.^1^ As a result, the 3D printing of models has ceased to be an isolated innovation and has become a common practice in the fabrication of orthodontic appliances, such as aligners.^2^ ^,^ ^3^

In the field of Dentistry, conventional impression techniques with plaster model fabrication are still widely used and offer good practical applicability. However, the technique is sensible and requires significant clinical control to ensure quality impressions. Additionally, plaster models present several disadvantages, such as lengthy processing time, potential risk of fractures, and the need for extensive storage space.^4^ ^,^ ^5^ With the introduction of intraoral scanning, it has become possible to digitally store patient data in STL files, which can be converted into physical printed models on demand, optimizing time, space, and resources.^6^ ^-^ ^8^

The subsequent fabrication of physical models can be performed using additive or subtractive methods and is carried out only when necessary. In this way, digital treatment planning offers a more efficient workflow, requiring less manual labor and potentially saving both time and physical space.^7^ ^,^ ^8^

The American Society for Testing and Materials (ASTM) has defined additive manufacturing (AM) as a process of joining materials to make objects from 3D model data, layer upon layer, as opposed to subtractive manufacturing methodologies.^9^ ^,^ ^10^ This technology is already widely used in Orthodontics, particularly in the production of models for the fabrication of aligners, responding to the need for accurately recording the configuration of dental movements and relationships.^4^ ^,^ ^6^

Orthodontic treatment using aligners requires a high level of precision for each appliance. During their manufacturing process, aligners are thermoformed over dental models; therefore, the accuracy of the 3D-printed models is essential for the success of the treatment.^11^ However, studies indicate that several factors can affect the dimensional stability of these models, including storage time and conditions - such as light exposure, temperature, and humidity - as well as the type of printer and the material used in the printing process.^12^ ^-^ ^14^ Still, there is a shortage of studies specifically analyzing the stability of printed models over time under real clinical conditions, especially for the fabrication of orthodontic aligners. This gap is relevant because model deformation can lead to poorly fitting aligners, compromising the effectiveness of tooth movement and interfering with the course of treatment.

The available literature on the stability of 3D-printed models lacks standardization regarding printing parameters and storage conditions, making it difficult to accurately assess the influence of storage time. Therefore, the aim of the present study was to evaluate the dimensional stability of 3D-printed models used for the fabrication of orthodontic aligners, using only one type of resin and storing the models in a light-free environment for a period of up to six months.

## MATERIAL AND METHODS

**Figure 1:**
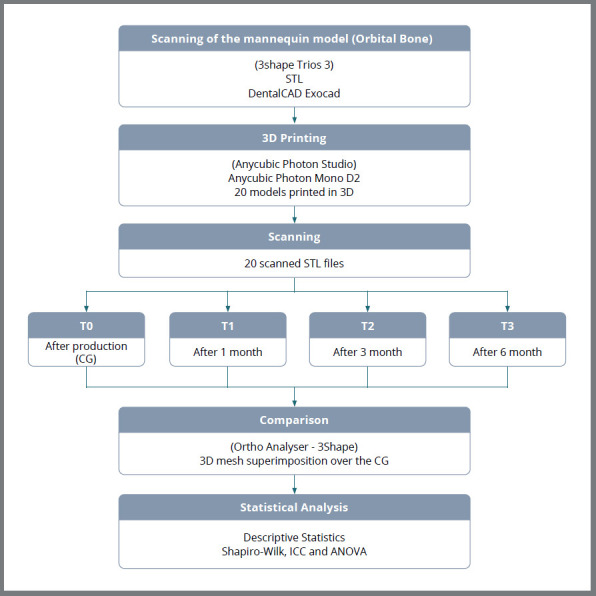
Flowchart of the study design (CG = control group).

The flowchart with the design of the present *in vitro* study is shown in Figure 1. A model of the upper arch of a dental mannequin (Orbital Bone) was scanned using a scanner (3Shape, Trios 3), and the digital model was exported in Standard Tessellation Language (STL) format to the DentalCAD software (Exocad) with the purpose of creating a model for printing. Three circular reliefs (or stops) were created on the model. These were strategically positioned to allow triangulation and subsequent superimposition of digital models. Anatomically, the points were located as follows: one on the midline over the incisive papilla region and the other two bilaterally, adjacent to the palatal marginal ridge of the first permanent molars.

The sample size was calculated based on the comparison of dimensional changes, assuming an estimated standard deviation of the difference of 0.3 mm, a minimum detectable difference of 0.2 mm, α = 5%, and power (1-β) = 80%.^15^ The number of models calculated for the sample was 19, and it was decided to use 20 models.

The height and thickness of the specimens were determined similarly to the method used for fabricating models for orthodontic aligners, using a standard layer thickness of 50 µm and a height of 2.5 cm, taking as reference the distance from the gingival zenith of the central incisor to the base of the model.

The models were numbered and prepared for printing using the Anycubic Photon Studio software (Anycubic Technology Co., Ltd., Shenzhen, China), and subsequently printed on a single printer (Anycubic Photon Mono X6 KS, Anycubic Technology Co., Ltd., Shenzhen, China), using Anycubic Water Wash Resin + (Shenzhen, China). Finally, they were washed with water using the Wash and Cure Plus device (Anycubic, Technology Co., Ltd., Shenzhen, China), subjected to a post-curing process under ultraviolet light for 15 minutes.

Scanner calibration was performed before the beginning of the study using the resin calibrator from Makertech (Makertech Labs, São Paulo, Brazil), and all scans were carried out by a trained and experienced professional. The models, kept in a dark box at room temperature (25°C), were scanned at the following time intervals: T0 - immediately after printing (control group), T1 - 1 month, T2 - 3 months, and T3 - 6 months. The digitized meshes of the models were superimposed over the control group using the Ortho Analyser software (3Shape, Copenhagen, Denmark), applying the three-surface alignment and triangulation method.

**Figure 2:**
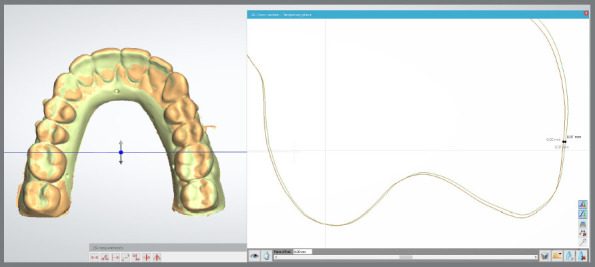
Triangulation-based superimposition of STL file meshes with a coronal section at the mesiobuccal cusp of tooth #26, using the Orthoanalyser software.

The differences between the meshes were blindly evaluated in the following regions of the dental arch: incisors, canines, premolars, and molars. To quantify the changes, a coronal section was made at the center of the incisal edge of the incisors, the cusp tip of the canines and premolars, and the mesiobuccal cusp of the molars, allowing the analysis of possible changes through two points selected, with the aid of the software, in the middle thirds of the teeth (Fig. 2). The 3D superimpositions of the meshes were performed in the Orthoanalyser software (3Shape Ortho System 2023) and visualized in color maps using a 0.1-mm scale, enabling the identification of possible changes and the understanding of dimensional variations.

The data obtained during the process were organized and transferred to spreadsheets in Microsoft Excel (Microsoft Corporation, Redmond, Washington, United States) for subsequent statistical analysis. The training and calibration of the sole examiner were conducted by an orthodontic specialist with experience in digital workflow.

## STATISTICAL ANALYSIS

To assess intra-examiner agreement and determine the reliability of the method, the differences between the meshes after 3D superimposition were measured twice, with a 15-day interval, covering 50% of the sample selected randomly. The analysis was performed using the Intraclass Correlation Coefficient (ICC), based on the following interpretation criteria: ICC < 0.4 (poor), 0.4 to 0.6 (fair), 0.6 to 0.75 (good), and 0.75 to 1.0 (excellent).^16^

Regarding the dimensional changes in the digital models, the data were initially subjected to the Shapiro-Wilk normality test, which showed the value: p > 0.05 for all parameters analyzed, validating the use of parametric tests. Thus, descriptive analysis was performed using the mean and standard deviation, followed by one-way Analysis of Variance (ANOVA) to determine the influence of the storage time variable (T1, T2, and T3) on the dimensional changes of the models. Data were analyzed using the BioEstat software (version 5.3, Mamirauá Institute, Tefé, Amazon, Brazil), and results were considered statistically significant at p < 0.05.

## RESULTS

The intra-examiner agreement in measuring the parameters used in the present study was characterized as excellent, with coefficients ranging from 0.84 to 0.96.

**Figure 3:**
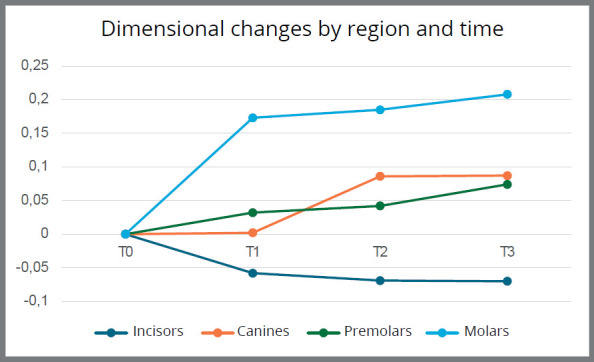
Mean dimensional variation by dental region over time (T1 = 1 month, T2 = 3 months, T3 = 6 months). Values refer to the difference in relation to the initial time (T0).

The dimensional changes in the 3D printed models are presented in Table 1. The results did not show any statistically significant dimensional changes at any of the model storage intervals, regardless of the region evaluated in the superimposition - whether anterior or posterior in the arch (p > 0.05). The greatest dimensional changes occurred within the first month, except in the canines, and were most pronounced in the molar region, which showed an expansion of 0.173 mm in the first month (T1), reaching 0.208 mm after six months (T3) of storage (Fig. 3).

**Table 1:** Descriptive analysis, confidence interval, and p-value for the 3D superimpositions in the incisor, canine, premolar, and molar regions according to the storage time.

Overlap	n	Storage Time									P
		1 month (T1-T0)			3 month (T2-T0)			6 month (T3-T0)			
		Average (mm)	Standard deviation	Confidence Interval	Average (mm)	Standard deviation	Confidence Interval	Average (mm)	Standard deviation	Confidence Interval	
Incisors	20	-0.058	0.083	-0.020 - -0.096	-0.069	0.069	-0.101 - -0.037	-0.070	0.092	-0.113 - -0.027	0.62
Canines	20	0.002	0.166	-0.075 - 0.079	0.086	0.153	0.015 - 0.157	0.087	0.150	0.017 - 0.157	0.95
Premolars	20	0.032	0.098	-0.013 - 0.077	0.042	0.104	-0.006 - 0.090	0.074	0.111	0.023 - 0.125	0.73
Molars	20	0.173	0.175	0.092 - 0.254	0.185	0.176	0.103 - 0.267	0.208	0.216	0.107 - 0.309	0.96

**Figure 4:**
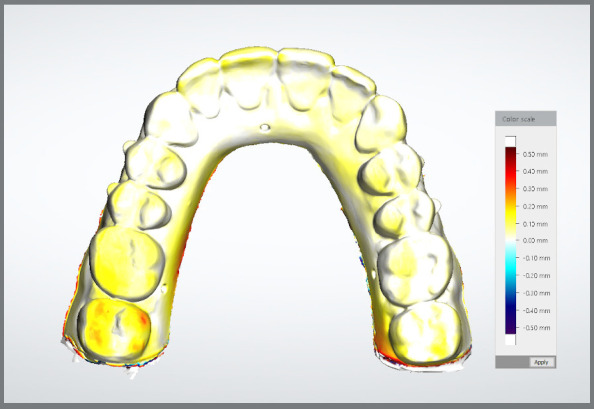
3D superimpositions of the meshes (control group × 6 months of storage) visualized in color maps using a 0.1-mm scale. White indicates no change, yellow indicates a 0.1-mm change, and orange indicates a 0.2-mm change.

However, the pattern of dimensional changes was different in the incisors, which tended to show a slight contraction (−0.07 mm), compared to the other regions of the arch, which exhibited expansion. The 3D color map (Fig. 4) highlighted the magnitude of the changes observed throughout the study.

## DISCUSSION

The evolution of 3D technology in modern Dentistry has enabled the development of more predictable and accessible procedures for both patients and, in Orthodontics, the digital workflow has shown wide applicability, enabling virtual treatment planning and the production of 3D models for the fabrication of aligners and other appliances, as it allows high-resolution resin printing from digitally stored data.^6^ In this *in vitro* study, the dimensional stability of 3D resin-printed models was evaluated over different storage time intervals, to determine their applicability in orthodontic aligner treatments.

The total sample of 20 3D-printed models was subjected to standardized conditions for both storage, kept in a light-free environment at room temperature (25°C), and printing, exclusively using stereolithography (SLA) printing technology (Anycubic Photon X6 KS) and resin (Anycubic Water Wash Resin +). Recent studies have indicated that printed materials exhibit less dimensional change when kept in a dark environment at room temperature, compared to exposure to light or to very low or very high temperatures.^17^ ^,^ ^18^ It is also worth noting that dimensional changes are related to the type of resin, with low-viscosity resins containing monomers with low shrinkage being recommended.^19^ Therefore, the conditions adopted in the present study were considered ideal for storage.

Although the available literature on the dimensional stability of 3D printed materials over time also highlights deviations of limited magnitude, some studies suggest the possibility of clinically relevant implications,^15^ ^,^ ^20^ while others consider model storage to be safe.^14^ ^,^ ^17^ ^,^ ^18^ The methodological heterogeneity, combined with the wide variety of storage conditions, materials, and types of printer used, complicates the comparison of results.

However, although the results of this study show no statistically significant dimensional changes for up to six months, the clinical implications of long-term storage remain unclear.^15^ The stability of dental models over time has already been a topic of scientific interest in previous experiments and studies related to 3D printing.^12^ ^,^ ^14^ ^,^ ^17^ ^,^ ^21^ The findings are consistent with the results of this research, which demonstrated a tendency for dimensional changes to increase as the storage time extended, with the magnitude of changes being greatest during the first month of storage.

The superimposition of the models showed that the pattern of dimensional changes consisted of a slight arch expansion, except in the incisor region, where a tendency for contraction was observed (−0.058 mm at T1 to −0.069 mm at T3). Arch expansion was greatest in the molar region, ranging from 0.175 mm at T1 to 0.208 mm at T3, while in the canine and premolar regions, the expansion was less than 0.1 mm. Similarly, the investigation by Rungrojwittayakul et al.^22^ indicated a heterogeneous pattern of variations from the time of printing, with contraction and expansion occurring in different areas of the models. In agreement with the study by Yousef et al.^17^, the results indicated that deformations of the printed models were not limited to linear expansion, which may also be related to the bilateral cantilever printing position not connected by a bar. A study with a five-month storage period reported a model contraction of approximately 0.26 mm, greater in the group exposed to light, but did not specify whether this deformation was uniform across all regions of the dental arch,^15^ making comparison of the results difficult.

In orthodontic treatment, aligners are virtually planned using algorithms to move teeth by 0.15-0.30 mm every two weeks.^23^ Based on this average movement per aligner, the findings of the present study suggest that the dimensional changes observed over a storage period of up to six months could have some clinical impact, particularly in the molar region, where an average expansion of 0.2 mm was observed. One strategy to minimize dimensional changes at the ends of the arch would be the fabrication of inter-molar bars during the preparation of digital models, prior to printing.^24^

The environmental impact associated with aligners is a sustainability concern, resulting from the excess residual material,^25^ ^-^ ^27^ which includes a large number of printed resin models and polymers that release a variety of nanoplastics.^28^ In this context, storing printed models, allowing their reuse in cases of aligner loss or breakage, could be beneficial, as it would reduce costs and be more environmentally friendly. However, our results suggest that further studies are needed to evaluate long-term storage, since storing printed models for up to six months requires caution, not only because of possible dimensional changes inherent to the material, but also due to small dental movements or micro-traumas that may occur during this period -factors that can compromise the precise fit of aligners. In the case of acetate thermoformed retainers, it is recommended to perform new scans annually, as the continuous process of intercuspation may alter the occlusal relationship and affect the stability and fit of the device.

## CONCLUSION

After six months of storage in a light-free environment, the 3D-printed models exhibited minor dimensional changes. Although small, these changes may have clinical relevance, particularly in the molar region, and should be considered in the planning and execution of orthodontic treatments with aligners.

## Data Availability

All data generated or analyzed during this study are included in this published article.
